# Obesity and Comorbidities in HFpEF: A Retrospective Cohort Analysis in a University Hospital Setting

**DOI:** 10.3390/jcm14103348

**Published:** 2025-05-12

**Authors:** Anastasia Janina Hobbach, Tobias Johannes Brix, Veronika Weyer-Elberich, Julian Varghese, Holger Reinecke, Wolfgang Albrecht Linke

**Affiliations:** 1 Department of Cardiology I, Coronary, Peripheral Vascular Disease and Heart Failure, University Hospital Münster, 48149 Münster, Germany; holger.reinecke@ukmuenster.de; 2 Institute of Physiology II, University of Münster, 48149 Münster, Germany; 3 Institute of Medical Informatics, University of Münster, 48149 Münster, Germany; tobias.brix@uni-muenster.de (T.J.B.); julian.varghese@uni-muenster.de (J.V.); 4 Institute of Biostatistics and Clinical Research, University of Münster, 48149 Münster, Germany; veronika.weyer-elberich@ukmuenster.de

**Keywords:** HFpEF, obesity, sex, body mass index, Germany, real-world data, comorbidities

## Abstract

**Background/Objectives**: Heart failure (HF) with preserved ejection fraction (HFpEF) poses a diagnostic challenge, as it lacks a definitive hallmark beyond preserved left ventricular (LV)EF. This retrospective study aims to analyze the demographic and clinical characteristics of HFpEF patients within a real-world hospital cohort, with a particular focus on obesity and associated comorbidities. **Methods**: A total of 4019 patients who underwent echocardiography in 2020–2021 at a university hospital were screened for HFpEF. After stringent manual verification, 219 patients fulfilled the European Society of Cardiology (ESC) criteria for HFpEF. Demographic, clinical, and comorbidity data were analyzed and stratified by body mass index (BMI) categories and sex distribution. **Results**: Among the 219 HFpEF patients, 71.3% were classified as pre-obese or obese. Hypertension (93.6%), atrial fibrillation (74.3%), and obesity were common in the cohort, while sex distribution was balanced. Edema was more prevalent among obese patients, though HFpEF severity, as reflected by New York Heart Association (NYHA) classification and natriuretic peptide levels, did not differ significantly across BMI groups. Medical treatment patterns varied with BMI, with obese patients more frequently receiving diuretics, sodium-glucose cotransporter-2 inhibitors (SGLT-2i), and angiotensin receptor blockers (ARB). No significant differences were observed between male and female patients in terms of HFpEF severity markers. **Conclusions**: Obesity is a predominant feature in HFpEF and is associated with a high burden of comorbidities. However, sex does not appear to influence HFpEF severity in this cohort. These findings underscore the need for targeted therapeutic strategies in obese HFpEF patients.

## 1. Introduction

Heart failure (HF) with preserved ejection fraction (HFpEF) is a common yet complex condition, representing a substantial proportion of heart failure cases worldwide. Unlike HF with reduced ejection fraction (HFrEF), HFpEF lacks a single defining diagnostic criterion beyond preserved LVEF (≥50%) [1,2]. Diagnosis is further complicated by the heterogeneity of HFpEF phenotypes, overlapping symptoms with other conditions, and the need for multimodal assessment strategies, including echocardiographic and biomarker-based evaluations. This complexity has contributed to ongoing challenges in both clinical management and research.

Among various risk factors for HFpEF, obesity has emerged as a significant contributor [3,4,5,6,7]; it has been incorporated into established diagnostic tools such as the H_2_FPEF score [8]. Adiposity exerts pathophysiological effects on cardiac structure and function through multiple mechanisms, including increased circulating blood volume and cardiac output, systemic inflammation, endothelial dysfunction, insulin resistance, and neurohormonal activation [7,9,10]. These factors collectively promote left ventricular hypertrophy, impaired diastolic relaxation, and increased myocardial stiffness [7,9,10]. Obesity-related comorbidities, such as diabetes and obstructive sleep apnea, further exacerbate cardiac dysfunction, contributing to the emergence of a distinct “obese-HFpEF” phenotype [11,12]. Moreover, adipose tissue-derived cytokines (adipokines) are believed to directly modulate myocardial remodeling, favoring a pro-fibrotic environment that leads to impaired ventricular compliance and elevated filling pressures [7,9,10].

While obesity and its metabolic consequences have a strong association with HFpEF, the independent impact of sex remains controversial [13,14]. HFpEF was thought to disproportionately affect women, but emerging evidence suggests that the strong link between obesity and HFpEF may confound sex-specific differences [13,14,15]. Recent studies indicate that the rising prevalence of obesity in both sexes may have reshaped the classical HFpEF demographic profile [15].

The objective of this study was to systematically characterize the demographic, clinical, and comorbidity profiles of HFpEF patients diagnosed in a real-world university hospital setting. Special weight was placed on analyzing the prevalence of obesity within this cohort, exploring its association with disease severity and clinical characteristics. Additionally, we sought to examine potential sex-based differences in HFpEF presentation. This study aims to inform future diagnostic and therapeutic strategies tailored to the metabolic HFpEF phenotype.

## 2. Materials and Methods

### 2.1. Selection and Analysis of the Study Population

We used a retrospective approach to characterize HFpEF patients in a real-world setting. This study adheres to the principles outlined in the Declaration of Helsinki (Br med J 1964, ii: 177). The research protocol received approval from the locally appointed ethics committee (Ethik-Kommission der Ärztekammer Westfalen-Lippe und der Universität Münster) under the approval number 2022-146-f-S.

Patient selection proceeded through multiple stages. Initially, we utilized Structured Query Language (SQL) to screen the electronic medical records database at University Hospital Münster (UKM), Germany, for medical reports/doctor’s letters to identify potential HFpEF cases during 2020–2021. The algorithm searched for the term “HFpEF”. Due to inconsistencies in HFpEF diagnosis and documentation, this initial approach yielded only a small number of potential HFpEF candidates (N = 183). Therefore, we adjusted our approach and let the algorithm search for patients visiting the Department of Cardiology at UKM between 2020 and 2021 under the term ‘HFpEF’ or featuring echocardiographic criteria of ‘LVEF ≥ 50%’ and ‘no previous LVEF < 45%’. This approach successfully identified many more cases, yielding 4.019 potential HFpEF candidates. All records identified through this automated process were then manually screened to determine whether they satisfied the latest diagnostic criteria (2020) for HFpEF based on the HFA/ESC diagnostic algorithm [2]. This screening approach revealed 219 patients with confirmed HFpEF. Patients who had undergone heart transplantation, had specific cardiomyopathies, or had congenital heart disease were excluded from the study.

Demographic information and available medical findings were gathered from eligible patients. The data were extracted from the electronic medical records; no new diagnoses were established apart from HFpEF. Only previously coded or listed diagnoses were incorporated. Regarding NYHA classification, we adopted the NYHA stages as documented by the treating physicians: in the clinical records, NYHA stages were often noted as intermediate (e.g., II–III), indicating variability in symptom severity. Given the retrospective and descriptive nature of the study, we did not attempt to retrospectively reclassify NYHA stages, as a reliable reassessment of symptom burden was not feasible. Medical records included (i) demographic details, such as age, body mass index (BMI), sex (assigned based on the information in the electronic medical record), and blood pressure (systolic/diastolic); (ii) comorbidities including atrial fibrillation (AF), arterial hypertension (AHT), diabetes, (former) nicotine abuse, cerebrovascular disease (CVD), chronic kidney disease (CKD), peripheral artery disease (PAD), coronary heart disease (CHD), myocardial infarction (MI), stroke/transient ischemic attack (TIA), chronic obstructive pulmonary disease (COPD), sleep apnea, or pulmonary arterial hypertension (PAH); (iii) clinical assessments like New York Heart Association (NYHA) class, edema, H_2_FPEF and HFA-PEFF score; (iv) laboratory findings for sodium, potassium, urea, creatinine, estimated glomerular filtration rate (eGFR), N-terminal brain natriuretic peptide (NT-proBNP), hemoglobin, hematocrit, and glycated hemoglobin (HbA1c); (v) prescribed medical therapies including angiotensin-converting enzyme inhibitor (ACEi), angiotensin receptor blocker (ARB), beta blocker, angiotensin receptor–neprilysin inhibitor (ARNI), calcium channel blocker (CCB), diuretics, mineralocorticoid receptor antagonist (MRA), sodium glucose cotransporter-2 inhibitor (SGLT-2i), acetylsalicylic acid (ASA), statins, and oral anticoagulants (OACs); (vi) the presence of implanted interatrial shunt device (IASD); (vii) echocardiographic findings, such as left atrial volume index (LAVI), E/A, e’ septal, e’ lateral, E/e’, degree of diastolic dysfunction, and LVEF; and (viii) invasive hemodynamic measurements (all conducted at rest) like cardiac index (CI) and pulmonary capillary wedge pressure (PCWP). Systolic HF therapy was defined as the use of at least three of the four ‘Fantastic Four’ medications (ACEi/ARB/ARNI, beta blocker, MRA, SGLT-2i) with or without the use of diuretics. At this point, it should be noted that, on occasion, ARNIs and MRAs have also been prescribed as an off-label therapeutic trial in cases of preserved EF and therapy-refractory dyspnea. Furthermore, some medications commonly prescribed for HFrEF may also have been administered due to comorbidities, for example, beta-blockers in the context of AF. Data were collected for each patient and for each of their visits throughout the years 2020–2021.

Statistical analyses were conducted using data from the patients’ most recent visit during this period. In instances of missing data, information was retrieved from previous visits in 2020–2021, except for NT-proBNP levels: the most recent NT-proBNP values were utilized even if measured before 2020. NT-proBNP levels measured during acute coronary syndrome were omitted. If NT-proBNP measurements were conducted externally rather than at UKM, these data were included in the analysis, given the diagnostic significance of NT-proBNP for the HFA-PEFF score.

Obesity and female sex were regarded as key factors. As for obesity, HFpEF patients were categorized into three groups based on their BMI: category I with BMI < 25 kg/m^2^, category II with BMI 25–30 kg/m^2^, and category III with BMI > 30 kg/m^2^.

### 2.2. Statistical Analysis

Sociodemographic and disease-related data underwent evaluation utilizing descriptive statistics. Patient cohorts were delineated by absolute numbers and percentages for categorical variables, and by mean with standard deviation (SD) or median with quartile range for continuous variables. A confidence interval of 0.95 was applied and tested as a complete case analysis with pairwise deletion for missing data. Normality assessment was conducted via Kolmogorov–Smirnov or Shapiro–Wilk test.

Group differences for non-normally distributed values were scrutinized using Kruskal–Wallis tests for BMI categories and Mann–Whitney-U tests for sex. For normally distributed outcome values, Student’s t-test was executed to compare male and female patients and a one-way analysis of variance (ANOVA) among patients across various BMI categories. All analyses were explorative; nevertheless, *p*-values < 0.05 were considered significant, with the caveat that these should be interpreted very cautiously in the context of an exploratory approach and multiple testing. No corrections for multiple comparisons were performed, except for the pairwise post hoc analyses from ANOVA using the Bonferroni test. The confidence interval was set at 0.95, and missing data were pairwise deleted test by test. *p*-values were provided solely for descriptive and explorative purposes and should be interpreted cautiously. The correlation between two ordinal variables was computed using Spearman’s rank-order, while the correlation among two categorical variables was assessed via Pearson or Kendall Tau correlation. All statistical analyses were conducted using the Statistical Package for the Social Sciences (SPSS, Chicago, IL, USA, version 28.0.1.1).

### 2.3. Data Statement

As our data include sensitive information (patient data), they are unavailable to access.

## 3. Results

An initial SQL screening of patients’ medical records using the term ‘HFpEF’– without distinguishing between confirmed or excluded HFpEF cases—yielded a limited number of instances (N = 183). Consequently, we expanded our search criteria, supported by the HFA/ESC recommendations of 2020 [2], to also include the echocardiographic parameters of “LVEF ≥ 50%” and “no previous LVEF < 45%”. This automated SQL screening process expanded the cohort of eligible patients to 4019 out of 5595 patients who received an echocardiography during the observation period at our hospital. These patients were then reviewed to confirm whether they reported dyspnea (at least on exertion). This was confirmed for all cases. Subsequently, all medical records were manually searched to determine whether “HFpEF had already been diagnosed”, or “HFpEF could be confirmed using the H_2_FPEF or HFA-PEFF scores”. Ultimately, 219 patients met the HFpEF criteria (many were “lost” because relevant data were not documented). Their average age was 75.03 ± 9.16 (mean ± SD) years, with an equal distribution of male and female patients.

### 3.1. Limited HFpEF Case Identification with the Need for Methodological Adjustments

**Figure 1:**
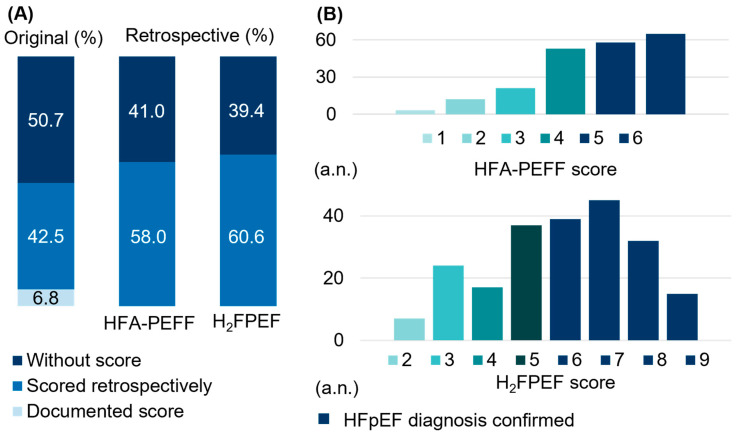
Retrospective HFpEF diagnosis in our cohort. (**A**) In 2020–2021, 219 HFpEF cases were verified at our center, out of which 50.7% of patients were previously diagnosed with HFpEF without using a HFpEF scoring system, and a HFpEF score was documented in 6.8%. In 42.5% of patients, HFpEF was diagnosed retrospectively in our study using the HFA-PEFF score (left). The retrospective application of the HFA-PEFF score to all patients would have allowed for the diagnosis of HFpEF (5 to 6 points) in 58.0% of patients without requiring invasive measurements (middle). The retrospective application of the H2FPEF score indicated a > 90% likelihood of HFpEF (minimum of 6 points) in 60.6% of cases. (**B**) The distribution of HFA-PEFF (top) and H2FPEF (bottom) score points in the study cohort. The dark blue color indicates confirmed HFpEF cases. a.n., absolute numbers.

None of the 219 patients identified with HFpEF had a documented HFA-PEFF score, and only 6.8% (N = 15) had a recorded H_2_FPEF score (Figure 1; Table 1). In 42.5% (N = 93) of cases, the diagnosis of HFpEF was unknown at the time of study inclusion but could be provided retrospectively using the HFA-PEFF score.

These findings illustrate a striking gap in the systematic application of HFpEF diagnostic criteria, even in an academic cardiology center. Despite being a specialized setting, nearly half of HFpEF cases had initially gone unrecognized, stressing the limitations of routine clinical diagnostics.

Moreover, 50.7% (N = 111) of HFpEF cases were previously diagnosed without documented scores (e.g., via invasive measurements (PCWP ≥ 15 mmHg)). Retrospectively, based on the available medical findings from 2020 to 2021, HFpEF could have been diagnosed in 58.02% (N = 123) of cases using the HFA-PEFF score (HFA-PEFF ≥ 5) without necessitating invasive measurements (Figure 1; Table 1). Furthermore, in 60.6% (N = 131) of patients, the retrospectively applied H_2_FPEF score was ≥6, indicating a likelihood of HFpEF exceeding 90% (Figure 1; Table 1). For the diagnosis of HFpEF, we followed the HFA-PEFF algorithm, which uses natriuretic peptide cut-offs based on rhythm status (sinus rhythm vs. AF) but does not apply age-adjusted thresholds [1]. Using recently proposed age-adjusted NT-proBNP thresholds [16], 18 patients would have exhibited NT-proBNP values within the normal range; among them, 9 patients had already shown normal NT-proBNP levels according to the HFA-PEFF algorithm, whereas 9 additional patients would have been reclassified as normal solely when applying the age-adjusted thresholds.

These results emphasize the challenges of HFpEF identification in real-world clinical practice and the necessity of structured, score-based methodologies to improve case detection and diagnostic accuracy.

### 3.2. Obesity and Comorbidities

The average BMI in our cohort (N = 216) was 28.80 ± 6.01 kg/m^2^ (mean ± SD), indicating a pre-obese classification with substantial variability. We categorized HFpEF patients into three groups based on BMI: under-to-normal weight (BMI < 25 kg/m^2^, 28.7%, N = 62), pre-obese (BMI 25 to 30 kg/m^2^, 30.1%, N = 65), and obese (BMI > 30 kg/m^2^, 41.2%, N = 89) (Table 2), assigning 154 patients (71.3%) to the pre-obese or obese categories.

Hypertension (93.6%) and atrial fibrillation (74.3%) were the most common comorbidities, while diabetes (35.6%) and chronic kidney disease (55.7%) were also frequently observed. Although a slight increase in the prevalence of comorbidities and edema was noted in obese patients, these observations do not imply significant correlations. Overall, edema was more common in obese patients (Table 2), though HFpEF severity indicators such as NYHA class and natriuretic peptide levels did not significantly vary across BMI categories. Obese HFpEF patients were generally younger than those in the lower-BMI categories I and II, and conditions such as diabetes and sleep apnea were more prevalent in these patients (Table 2). However, statistical analysis revealed weak correlations between BMI and age, NYHA class, or edema: the Pearson correlation coefficients were *r* = −0.201 for age and BMI, *r* = 0.153 for NYHA class and BMI, and *r* = 0.233 for edema and BMI, respectively (Table 2 and Table S1).

Medical therapy also varied by BMI category, with more a frequent subscription of ARB (BMI I > III), ARNI (BMI III > II), CCBs (BMI III > I), diuretics (BMI III > I; III > II), and SGLT-2i (BMI III > I; II > I) in patients with higher BMI (Table 2). Obese patients more frequently received a more comprehensive treatment regimen for systolic HF (*p* < 0.001 for BMI III vs. I) (Table 2).

### 3.3. Female Vs. Male Sex in HFpEF Symptom Severity

With half of the patients being female, we investigated possible sex-related disparities in the data. As anticipated, significant variation was observed regarding creatinine levels (m > f, *p* = 0.022), hemoglobin (m > f, *p* = 0.042), and hematocrit (m > f, *p* = 0.036) (Table 3). Male patients showed a higher prevalence of (former) nicotine abuse (*p* < 0.001) and a greater number of comorbidities (*p* = 0.002) compared to females, including CVD (*p* = 0.007), PAD (*p* = 0.036), CHD (*p* = 0.009), stroke (*p* = 0.028), and sleep apnea (*p* = 0.022) (Table 3). Furthermore, the E/e’ ratio as an indicator of diastolic dysfunction was significantly higher in females than males (*p* = 0.006) (Table 3). No significant differences were found between the sexes with regard to BMI (*p* = 0.397) or parameters indicating HFpEF severity, including edema (*p* = 0.146), median NYHA class (*p* = 0.066), H_2_FPEF (*p* = 0.850) and HFA-PEFF (*p* = 0.839) scores, NT-proBNP levels (*p* = 0.509), degree of diastolic dysfunction (*p* = 0.704), and CI (*p* = 0.305). Therefore, HFpEF severity in our cohort was largely independent of sex.

## 4. Discussion

Diagnosing HFpEF remains a major challenge in routine clinical practice, as evidenced by our study findings. Despite being conducted in an academic cardiology center—where diagnostic accuracy is expected to be high—approximately half of HFpEF cases were initially unrecognized. This features substantial gaps in standardized diagnostic application, even within specialized settings. A key factor contributing to this issue is the inconsistent use of established diagnostic scores: none of the 219 patients in our cohort had a documented HFA-PEFF score, and only 6.8% had a recorded H2FPEF score.

Our findings reinforce the broader challenges reported in the literature regarding the underdiagnosis of HFpEF [17]. Implementing systematic score-based approaches in clinical workflows could enhance recognition and lead to more accurate case identification. Additionally, broader educational initiatives targeting both general cardiologists and internal medicine specialists may help bridge the current diagnostic gaps.

Beyond diagnostic challenges, our study also emphasizes the significant role of obesity in HFpEF, as more than 70% of patients were classified as pre-obese or obese. The metabolic phenotype of HFpEF has already been emphasized by the inclusion of obesity as a diagnostic criterion in the H_2_FPEF score [4,5,6,18]. In our cohort, consistent with prior findings, obesity was associated with an increased burden of comorbidities, including hypertension and atrial fibrillation. The observed associations between obesity and both diabetes and sleep apnea further reinforce the metabolic phenotype of HFpEF, which has been increasingly recognized in recent research [3,4,5,6]. Our findings suggest that BMI as a marker for obesity itself may serve as a risk stratification tool in HFpEF. The significant differences in H_2_FPEF scores across BMI groups point toward a mechanistic link between adiposity and HFpEF severity. Given the established roles of inflammation, insulin resistance, and adipokine dysregulation in cardiac remodeling, these factors may contribute to the higher HFpEF burden in obese individuals.

The “obesity paradox”, whereby overweight and mildly obese individuals with HFrEF appear to have better survival outcomes than those with normal weight, has been extensively described [19,20]. However, it remains controversial whether this paradox applies to patients with HFpEF. Recent evidence suggests that overweight and obese patients with HFpEF may indeed exhibit a reduced risk of all-cause mortality compared to normal-weight individuals [21,22]. Nevertheless, while obesity is a key contributor to HFpEF pathophysiology through systemic inflammation, diastolic dysfunction, and myocardial remodeling, our study shows that the burden of comorbidities and clinical severity does not linearly correlate with BMI. These findings stress the complexity of obesity’s impact on HFpEF outcomes and hint that obesity-related phenotypes should be considered more holistically beyond BMI categorization alone.

NT-proBNP is known for its diagnostic and prognostic significance in HF; however, its utility as a consistent diagnostic tool in routine clinical practice is challenged by the fact that NT-proBNP levels can sometimes fall within normal ranges in cases of chronic HFpEF [23]. Moreover, NT-proBNP levels may also be influenced by age [16]. Although patients with HFpEF generally exhibit higher NT-proBNP levels than healthy individuals, those who are obese tend to display lower concentrations compared to non-obese HFpEFs [24], complicating the diagnostic process. Diagnosing HFpEF in obese patients is particularly challenging, not only due to technical limitations in imaging and the underestimation of structural abnormalities when indexed to body surface area, but also lower NT-proBNP levels. Echocardiographic techniques such as myocardial mass calculation, tissue Doppler imaging, mitral inflow assessment, and longitudinal strain analysis offer enhanced sensitivity for detecting subclinical diastolic dysfunction [7]. Cardiac magnetic resonance imaging and cardiac computed tomographic angiography are valuable adjuncts when echocardiographic imaging quality is poor. The systematic use of advanced multimodality imaging could improve the early diagnosis and risk stratification of HFpEF in obese individuals.

The higher medication intake observed in obese HFpEF patients reflects the complex challenge of managing patients with multiple morbidities. The treatment approach for HFpEF, influenced by comorbidities, does not align optimally with systolic HF trials [25]. A substantial proportion of patients receiving systolic HF treatment remained symptomatic, underscoring the need for specific therapeutic targets tailored for HFpEF. Recent advances in pharmacotherapy targeting metabolic pathways have introduced novel agents such as tirzepatide and semaglutide, which have shown promising results in reducing body weight and improving cardiometabolic profiles. These agents, particularly glucagon-like peptide-1 (GLP-1) receptor agonists and dual gastric inhibitory polypeptide (GIP)/GLP-1 receptor agonists, may offer new therapeutic avenues for obese patients with HFpEF, especially women who are disproportionately affected and often represent a distinct clinical cluster population [26,27,28]. Preliminary studies suggest that weight loss induced by these agents could not only reduce symptom burden but also favorably modulate cardiac structure and function, including improvements in ventricular remodeling and diastolic parameters [29]. Moreover, GLP-1 receptor agonists exert anti-inflammatory effects, which may be particularly beneficial in the inflammatory HFpEF phenotype frequently observed among obese female patients [30]. Although early clinical trials such as STEP-HFpEF and SUMMIT have provided encouraging data, robust long-term outcome studies in HFpEF populations, particularly regarding cardiovascular morbidity and mortality, are still awaited [26,28].

Sex-based differences in HFpEF remain a topic of debate. While previous studies have reported a higher incidence of HFpEF in women [13,14], our study did not observe a female predominance. Instead, both sexes were equally represented, with similar HFpEF severity markers. This aligns with emerging evidence suggesting that the historical female preponderance in HFpEF may be partially attributable to the high prevalence of obesity, which affects both sexes [15]. Notably, female sex is not recognized as a risk factor in either the HFA-PEFF or H_2_FPEF scores. It is arguable that the obesity pandemic has reshaped the classical HFpEF population to now include at least as many males as females. However, a comparison of “obesity” and “female sex” as independent factors for HFpEF in patients with and without the condition would be essential for a definitive understanding of their roles.

### Limitations

Our data capture the daily clinical reality at a university hospital in Germany. Consequently, our study has several limitations inherent to its retrospective design. First, the absence of complete echocardiographic data and NT-proBNP values, as well as the single-center setting, introduce potential bias. Second, since the study included patients visiting UKM in 2020–2021, it does not address the impact of more recent HFpEF treatment recommendations (SGLT-2i). Yet another limitation of our study is the challenge of excluding non-cardiac causes of dyspnea, such as COPD or pulmonary arterial hypertension, in a retrospective setting. Despite our efforts to minimize this bias by including only patients from a cardiology facility, we acknowledge that some cases may represent multifactorial dyspnea rather than isolated HFpEF.

The diverse patient population and the prevalence of patients without identification documents sometimes limited accurate age and (occasionally) even gender assignment. Furthermore, the retrospective nature of the study necessitated the retention of intermediate NYHA classes, as originally documented by treating physicians. Incomplete data for some patients, primarily due to variable clinical documentation, limited the implementation of complex statistical analyses, such as multivariate regression or propensity score matching. These missing data points posed challenges in fully assessing the robustness of our findings across all variables of interest.

We acknowledge that our screening approach, which focused on patients with preserved EF, may have introduced bias, as the common denominator was echocardiographic evidence of preserved EF rather than HF clinical syndrome. We thoroughly reviewed the medical records of patients to confirm the presence of HFpEF symptoms, and those without documented symptoms were excluded from the analysis. We are aware of the challenges in accurately assessing symptom burden retrospectively, and our approach may have overlooked some patients with HF symptoms which were not documented. Our study did not include a control group of non-HFpEF patients, such as healthy non-obese individuals or patients with HFrEF. This limits direct comparisons regarding the impact of obesity on HFpEF-specific characteristics. Follow-up studies should incorporate such control groups to enable a more comprehensive understanding of obesity-related pathophysiological mechanisms in HFpEF.

Future work with more comprehensive data-capture strategies could mitigate these limitations and enhance the validity of our results. Further research should also consider diverse patient demographics and treatment approaches in multicenter or population-based studies to provide a more thorough understanding of HFpEF and improve diagnostic and treatment strategies. Finally, standardized and prospective data collection protocols would help address the challenges posed by missing variables, such as blood pressure measurements, echocardiographic findings, and laboratory markers, which are critical for diagnostic scoring systems. These efforts would not only improve data completeness but also allow for a more robust evaluation of the diagnostic performance of non-invasive scores in broader clinical settings.

## 5. Conclusions

Our study draws attention to diagnostic challenges in HFpEF, even within specialized academic cardiology settings, underlining the need for a more consistent application of standardized diagnostic tools such as the HFA-PEFF and H2FPEF scores. The findings emphasize the substantial impact of obesity on HFpEF burden and severity, suggesting that body weight should be considered a key clinical factor when evaluating patients with suspected HFpEF. Additionally, the absence of a female predominance in our cohort contributes to the evolving understanding of HFpEF demographics in the context of rising obesity rates.

From a clinical practice perspective, our results advocate for more systematic and score-based diagnostic approaches, as well as heightened awareness of the metabolic profile in HFpEF patients. Improving the use of standardized diagnostic criteria may facilitate earlier recognition and more accurate classification of HFpEF, ultimately supporting more individualized patient management strategies. Future research should aim to validate these observations in larger, multicenter cohorts with prospective data collection to refine diagnostic processes and further characterize phenotypic differences within the HFpEF population.

## Figures and Tables

**Table 1:** Characteristics of patients with HFpEF in our cohort.

	Total (N = 219)
Demographic data	
Age—years—mean ± SD	75.03 ± 9.16 (N = 217)
Female—% (a.n)	50.0 (109) (N = 218)
Outpatients—% (a.n.)	60.5 (130) (N = 215)
BMI—kg/m^2^—mean ± SD	28.80 ± 6.01 (N = 216)
BMI category-median	II (N = 216)
BMI category—% (a.n.)	
I	28.7 (62)
II	30.1 (65)
III	41.2 (89)
BP (sys.)—mmHg—mean ± SD	138.22 ± 26.01 (N = 140)
BP (dias.)—mmHg—mean ± SD	78.76 ± 12.78 (N = 137)
Comorbidities	
AF—% (a.n.)	74.3 (162) (N = 218)
AHT—% (a.n.)	93.6 (205) (N = 218)
Diabetes—% (a.n.)	35.6 (78) (N = 218)
(ex-) nicotine abuse—% (a.n.)	33.0 (72) (N = 218)
CVD—% (a.n.)	12.4 (27) (N = 218)
PAD—% (a.n.)	18.3 (40) (N = 218)
CHD—% (a.n.)	43.6 (95) (N = 218)
MI—% (a.n.)	12.8 (28) (N = 218)
Stroke/TIA—% (a.n.)	13.3 (29) (N = 218)
COPD—% (a.n.)	18.3 (40) (N = 218)
Sleep apnea—% (a.n.)	14.7 (32) (N = 218)
PAH—% (a.n.)	21.6 (47) (N = 218)
CKD—% (a.n.)	55.7 (122) (N = 219)
Comorbidities—No.—mean ± SD	4.02 ± 1.72 (N = 218)
Clinical performance	
NYHA class median	II–III (N = 205)
NYHA class—% (a.n.)	
I	6.8 (14)
I–II	3.4 (7)
II	27.3 (56)
II–III	16.1 (33)
III	32.2 (66)
III–IV	6.3 (13)
IV	7.8 (16)
Edema—% (a.n.)	37.1 (73) (N = 197)
HFA-PEFF scoring total—median (IQR)	5.00 [2] (N = 212)
HFA-PEFF scoring—% (a.n.)	
1	1.4 (3)
2	5.6 (12)
3	9.9 (21)
4	24.2 (53)
5	27.2 (58)
6	30.5 (65)
H_2_FPEF scoring total—median (IQR)	6.00 [2] (N = 216)
H_2_FPEF scoring—% (a.n.)	
1	0.0 (0)
2	3.2 (7)
3	11.1 (24)
4	7.9 (17)
5	17.1 (37)
6	18.1 (39)
7	20.8 (45)
8	14.8 (32)
9	6.9 (15)
Laboratory findings	
Sodium—mmol/L—mean ± SD	139.78 ± 7.26 (N = 194)
Potassium—mmol/L—mean ± SD	4.40 ± 0.57 (N = 194)
Urea—mg/dL—mean ± SD	26.62 ± 15.21 (N = 156)
Creatinine—mg/dL—mean ± SD	1.44 ± 0.96 (N = 194)
eGFR—mL/min/1.73 m^2^—mean ± SD	50.28 ± 20.33 (N = 186)
NT-proBNP—pg/mL—median (IQR)	1261.00 (1776) (N = 186)
Hemoglobin—g/dL—mean ± SD	12.31 ± 1.95 (N = 194)
Hematocrit—%—mean ± SD	37.69 ± 6.43 (N = 194)
HbA1c—%—mean ± SD	8.6 ± 1.80 (N = 3)
Medical therapy	
ACEi—% (a.n.)	29.8 (65) (N = 218)
ARB—% (a.n.)	40.1 (87) (N = 217)
ARNI—% (a.n.)	6.0 (13) (N = 218)
ARNI/ACEi/ARB—% (a.n.)	75.1 (163) (N = 217)
Beta blockers—% (a.n.)	79.8 (174) (N = 218)
CCB—% (a.n.)	36.2 (79) (N = 218)
Diuretics—% (a.n.)	79.8 (174) (N = 218)
MRA—% (a.n.)	23.4 (51) (N = 218)
SGLT-2i—% (a.n.)	12.4 (27) (N = 218)
ASA—% (a.n.)	21.2 (46) (N = 217)
Statins—% (a.n.)	58.3 (127) (N = 218)
OAC—% (a.n.)	72.0 (157) (N = 218)
Sys. HF treatment—% (a.n.)	17.5 (38) (N = 217)
Device therapy	
IASD—% (a.n.)	6.4 (14) (N = 218)
Echocardiographic findings	
LVEF—%—mean ± SD	56.54 ± 5.41 (N = 218)
LAVI—mL/m^2^—mean ± SD	48.77 ± 18.92 (N = 185)
E/e’—mean ± SD	14.07 ± 5.35 (N = 135)
E/A— ± SD	1.78 ± 0.98 (N = 135)
e’ septal—cm/s—mean ± SD	7.10 ± 3.78 (N = 80)
e’ lateral—cm/s—mean ± SD	9.46 ± 3.31 (N = 86)
TR velocity—m/s—mean ± SD	3.12 ± 0.51 (N = 51)
Degree dias. dys.—total—median	II (N = 132)
Degree dias. dys.—% (a.n.)	
I	22.0 (29)
I–II	0.8 (1)
II	40.9 (54)
II–III	3.0 (4)
III	33.3 (44)
Invasive hemodynamics	
CI—L/min/m^2^—mean ± SD	2.35 ± 0.71 (N = 65)
PCWP—mmHg—mean ± SD	20.21 ± 7.28 (N = 61)

Average data shown as mean ± SD. Numbers followed by square brackets are median with interquartile range. Categorical variables are described by percentage and absolute number (a.n.). Colored rows indicate subcategories. ACEi, angiotensin-converting enzyme inhibitor; AHT, arterial hypertension; AF, atrial fibrillation; ARB, angiotensin receptor blocker; ARNI, angiotensin receptor–neprilysin inhibitor; ASA, acetylsalicylic acid; BMI, body mass index; BP, blood pressure; CCB, calcium channel blocker; CHD, coronary heart disease; CI, cardiac index; CKD, chronic kidney disease; COPD, chronic obstructive pulmonary disease; CVD, cerebrovascular disease; dias., diastolic; dys., dysfunction, eGFR, estimated glomerular filtration rate; HbA1c, glycated hemoglobin; HF, heart failure; IASD, implanted interatrial shunt device; IQR, interquartile range; LAVI, left atrial volume index; LVEF, left ventricular ejection fraction; MI, myocardial infarction; MRA, mineralocorticoid receptor antagonist; NT-proBNP, N-terminal pro-brain natriuretic peptide; NYHA, New York Heart Association; OACs, oral anticoagulants; PAD, peripheral artery disease; PAH, pulmonary arterial hypertension; PCWP, pulmonary capillary wedge pressure; SGLT-2i, sodium glucose cotransporter-2 inhibitor; sys., systolic; TIA, transient ischemic attack; TR, tricuspid valve regurgitation.

**Table 2:** BMI-dependent characteristics of our HFpEF cohort.

BMI Category	I (N = 62)	II (N = 65)	III (N = 89)	*p*-Value		
				I vs. II	I vs. III	II vs. III
Demographic data						
BMI—kg/m^2^—mean ± SD	22.37 ± 2.23 (N = 62)	27.29 ± 1.30 (N = 65)	34.39 ± 4.61 (N = 89)			
Age—years—mean ± SD	77.27 ± 7.60 (N = 62)	74.20 ± 10.41 (N = 65)	74.10 ± 9.05 (N = 88)	0.176	0.111	1.000
Female—% (a.n.)	58.1 (36) (N = 62)	47.7 (31) (N = 65)	46.1 (41) (N = 89)	0.734	0.447	1.000
Outpatients—% (a.n.)	59.7 (37) (N = 62)	57.1 (36) (N = 63)	65.5 (57) (N = 87)	1.000	1.000	0.906
BP (sys.)—mmHg—mean ± SD	135.42 ± 25.44 (N = 38)	137.65 ± 17.96 (N = 40)	138.92 ± 29.70 (N = 60)	0.394	0.868	0.437
BP (dias.)—mmHg—mean ± SD	77.53 ± 14.12 (N = 38)	81.51 ± 11.04 (N = 39)	77.24 ± 11.94 (N = 58)	0.085	0.901	0.077
Comorbidities						
AF—% (a.n.)	79.0 (49) (N = 62)	66.2 (43) (N = 65)	77.5 (69) (N = 89)	0.291	1.000	0.332
AHT—% (a.n.)	90.3 (56) (N = 62)	92.3 (60) (N = 65)	97.8 (87) (N = 89)	1.000	0.179	0.483
Diabetes—% (a.n.)	17.7 (11) (N = 62)	30.8 (20) (N = 65)	50.6 (45) (N = 89)	0.337	<0.001 *	0.027 *
(ex-)nicotine abuse—% (a.n.)	30.7 (19) (N = 62)	35.3 (23) (N = 65)	32.5 (29) (N = 89)	1.000	1.000	1.000
CVD—% (a.n.)	16.1 (10) (N = 62)	12.3 (8) (N = 65)	10.1 (9) (N = 89)	1.000	0.824	1.000
PAD—% (a.n.)	22.6 (14) (N = 62)	21.5 (14) (N = 65)	13.5 (12) (N = 89)	1.000	0.476	0.617
CHD—% (a.n.)	48.4 (30) (N = 62)	47.7 (31) (N = 65)	37.1 (33) (N = 89)	1.000	0.509	0.547
MI—% (a.n.)	12.9 (8) (N = 62)	16.9 (11) (N = 65)	10.1 (9) (N = 89)	1.000	1.000	0.651
Stroke/TIA—% (a.n.)	12.9 (8) (N = 62)	13.8 (9) (N = 65)	13.5 (12) (N = 89)	1.000	1.000	1.000
COPD—% (a.n.)	21.0 (13) (N = 62)	18.5 (12) (N = 65)	14.6 (13) (N = 89)	1.000	0.948	1.000
Sleep apnea—% (a.n.)	4.8 (3) (N = 62)	9.2 (6) (N = 65)	25.8 (23) (N = 89)	1.000	<0.001 *	0.011 *
PAH—% (a.n.)	22.6 (14) (N = 62)	16.9 (11) (N = 65)	23.6 (21) (N = 89)	1.000	0.963	1.000
CKD—% (a.n.)	58.1 (36) (N = 62)	49.2 (32) (N = 65)	58.4 (52) (N = 89)	0.958	1.000	0.779
Comorbidities—No.—mean ± SD	3.94 ± 1.62 (N = 62)	3.78 ± 1.68 (N = 65)	4.22 ± 1.80 (N = 89)	1.000	0.927	0.352
Clinical performance						
NYHA class median	II–III (N = 58)	II–III (N = 59)	III (N = 87)	0.962	0.065	0.652
NYHA class—% (a.n.)						
I	17.2 (10)	3.4 (2)	2.3 (2)			
I–II	1.7 (1)	8.5 (5)	1.1 (1)			
II	27.6 (16)	33.9 (20)	23.0 (20)			
II–III	10.3 (6)	11.9 (7)	23.0 (20)			
III	31.0 (18)	27.1 (16)	26.8 (32)			
III–IV	5.2 (3)	5.1 (3)	8.0 (7)			
IV	6.9 (4)	10.2 (6)	5.7 (5)			
Edema—% (a.n.)	19.0 (11) (N = 58)	38.6 (22) (N = 57)	47.5 (38) (N = 80)	0.079	0.002 *	0.827
HFA-PEFF scoring total-median (IQR)	5.00 [2] (N = 61)	5.00 [2] (N = 63)	5.00 [2] (N = 87)	1.000	1.000	1.000
HFA-PEFF sc.—% (a.n.)						
1	1.6 (1)	1.6 (1)	1.1 (1)			
2	4.9 (3)	3.2 (2)	8.0 (7)			
3	6.6 (4)	15.9 (10)	8.0 (7)			
4	27.9 (17)	15.9 (10)	29.9 (26)			
5	29.5 (5)	31.7 (20)	21.8 (19)			
6	29.5 (18)	31.7 (20)	29.9 (26)			
H_2_FPEF scoring total—median (IQR)	5.00 [2] (N = 61)	5.00 [4] (N = 64)	8.00 [2] (N = 89)	1.000	<0.001 *	<0.001 *
H_2_FPEF scoring—% (a.n.)						
1	0.0 (0)	0.0 (0)	0.0 (0)			
2	4.9 (3)	6.3 (4)	0.0 (0)			
3	14.8 (9)	21.9 (14)	1.1 (1)			
4	4.9 (3)	10.9 (7)	7.9 (7)			
5	29.5 (18)	15.6 (10)	10.1 (9)			
6	26.2 (16)	20.3 (13)	9.0 (8)			
7	19.7 (12)	21.9 (14)	21.3 (19)			
8	0.0 (0)	1.6 (1)	34.8 (8)			
9	0.0 (0)	1.6 (1)	15.7 (14)			
Laboratory findings						
Sodium—mmol/L—mean ± SD	140.55 ± 11.31 (N = 56)	138.58 ± 5.73 (N = 55)	140.06 ± 3.88 (N = 81)	0.466	1.000	0.738
Potassium—mmol/L—mean ± SD	4.35 ± 0.56 (N = 56)	4.38 ± 0.51 (N = 55)	4.45 ± 0.61 (N = 81)	1.000	0.824	1.000
Urea—mg/dL—mean ± SD	24.80 ± 14.52 (N = 46)	25.41 ± 15.80 (N = 43)	28.55 ± 15.38 (N = 66)	1.000	0.610	0.889
Creatinine—mg/dL—mean ± SD	1.33 ± 0.79 (N = 56)	1.51 ± 1.39 (N = 55)	1.47 ± 0.69 (N = 81)	1.000	1.000	1.000
eGFR—mL/min/1.73 m^2^—mean ± SD	53.39 ± 19.41 (N = 56)	51.76 ± 20.02 (N = 54)	47.31 ± 21.13 (N = 74)	0.707	0.066	0.156
NT-proBNP—pg/mL—median (IQR)	1464.50 (1757) (N = 54)	1139.50 (1542) (N = 56)	1215.00 (1973) (N = 74)	0.179	1.000	0.561
Hemoglobin—g/dL—mean ± SD	12.27 ± 1.78 (N = 56)	12.18 ± 1.82 (N = 55)	12.48 ± 2.13 (N = 81)	1.000	1.000	1.000
Hematocrit—%—mean ± SD	37.31 ± 4.85 (N = 56)	37.70 ± 8.33 (N = 55)	38.13 ± 5.93 (N = 81)	0.620	0.360	0.147
HbA1c—%—mean ± SD	0.00 (N = 0)	0.00 (N = 0)	8.6 ± 1.80 (N = 3)			
Medical therapy						
ACEi—% (a.n.)	33.9 (21) (N = 62)	36.9 (24) (N = 65)	21.3 (19) (N = 89)	1.000	0.291	0.110
ARB—% (a.n.)	29.0 (18) (N = 62)	38.5 (25) (N = 65)	48.9 (43) (N = 88)	0.828	0.044 *	0.577
ARNI—% (a.n.)	4.8 (3) (N = 62)	0.0 (0) (N = 65)	11.2 (10) (N = 89)	0.740	0.303	0.011 *
ARNI/ACEi/ARB—% (a.n.)	66.1 (41) (N = 62)	75.4 (49) (N = 65)	80.7 (71) (N = 88)	0.688	0.131	1.000
Beta blockers—% (a.n.)	79.0 (49) (N = 62)	80.0 (52) (N = 65)	80.9 (72) (N = 89)	1.000	1.000	1.000
CCB—% (a.n.)	25.8 (16) (N = 62)	32.3 (21) (N = 65)	47.2 (42) (N = 89)	1.000	0.021 *	0.17
Diuretics—% (a.n.)	72.6 (45) (N = 62)	72.3 (47) (N = 65)	89.9 (80) (N = 89)	1.000	0.027 *	0.021 *
MRA—% (a.n.)	14.5 (9) (N = 62)	23.1 (15) (N = 65)	29.2 (26) (N = 89)	0.758	0.107	1.000
SGLT-2i—% (a.n.)	0.0 (0) (N = 62)	15.4 (10) (N = 65)	18.0 (16) (N = 89)	0.021 *	0.002 *	1.000
ASA—% (a.n.)	24.2 (15) (N = 62)	20.0 (13) (N = 65)	19.3 (17) (N = 88)	1.000	1.000	1.000
Statins—% (a.n.)	58.1 (36) (N = 62)	55.4 (36) (N = 65)	61.8 (55) (N = 89)	1.000	1.000	1.000
OAC—% (a.n.)	67.7 (42) (N = 62)	69.2 (45) (N = 65)	77.5 (69) (N = 89)	1.000	0.567	0.775
Sys. HF treatment—% (a.n.)	6.5 (4) (N = 62)	18.4 (12) (N = 65)	23.9 (21) (N = 88)	0.135	<0.001 *	0.128
Device therapy						
IASD—% (a.n.)	4.8 (3) (N = 62)	4.6 (3) (N = 65)	9.0 (8) (N = 89)	1.000	0.933	0.837
Echocardiographic findings						
LVEF—%—mean ± SD	56.50 ± 4.40 (N = 62)	56.93 ± 5.62 (N = 65)	56.21 ± 5.89 (N = 89)	1.000	1.000	1.000
LAVI—mL/m^2^—mean ± SD	51.76 ± 21.09 (N = 53)	46.78 ± 16.34 (N = 57)	48.25 ± 19.23 (N = 74)	0.511	0.914	1.000
E/e’—mean ± SD	14.21 ± 5.80 (N = 45)	14.22 ± 4.92 (N = 54)	13.75 ± 5.42 (N = 64)	1.000	1.000	1.000
E/A—mean ± SD	1.59 ± 0.75 (N = 33)	1.77 ± 0.88 (N = 45)	1.88 ± 1.16 (N = 56)	1.000	0.517	1.000
e’ septal—cm/s—mean ± SD	5.85 ± 2.33 (N = 29)	6.53 ± 2.28 (N = 25)	9.20 ± 5.33 (N = 25)	1.000	0.003 *	0.029 *
e’ lateral—cm/s—mean ± SD	8.81 ± 3.13 (N = 31)	8.84 ± 2.72 (N = 25)	10.74 ± 3.71 (N = 29)	1.000	0.069	0.102
TR velocity—m/s—mean ± SD	3.00 ± 0.50 (N = 15)	3.08 ± 0.54 (N = 16)	3.17 ± 0.42 (N = 19)	1.000	0.985	1.000
Degree dias. dys.—total median	II (N = 38)	II (N = 40)	II (N = 53)	0.359	1.000	0.839
Degree dias. dys.—% (a.n.)						
I	26.3 (10)	15.5 (7)	22.6 (12)			
I–II	2.6 (1)	0.0 (0)	0.0 (0)			
II	42.1 (16)	35.0 (14)	45.3 (24)			
II–III	2.6 (1)	7.5 (3)	0.0 (0)			
III	26.3 (10)	40.0 (16)	32.1 (17)			
Invasive hemodynamics						
CI—L/min/m^2^—mean ± SD	2.24 ± 0.57 (N = 14)	2.58 ± 1.21 (N = 14)	2.28 ± 0.46 (N = 36)	0.655	1.000	0.551
PCWP—mmHg—mean ± SD	19.57 ± 5.95 (N = 14)	20.92 ± 12.87 (N = 12)	20.32 ± 5.16 (N = 34)	0.900	0.555	0.480

Average data shown as mean ± SD. Numbers followed by square brackets are median with interquartile range. Colored rows indicate subcategories. Significant differences are indicated by an asterisk. Categorical variables are described by percentage and absolute number (a.n.). ACEi, angiotensin-converting enzyme inhibitor; AHT, arterial hypertension; AF, atrial fibrillation; ARB, angiotensin receptor blocker; ARNI, angiotensin receptor–neprilysin inhibitor; ASA, acetylsalicylic acid; BMI, body mass index; BP, blood pressure; CCB, calcium channel blocker; CHD, coronary heart disease; CI, cardiac index; CKD, chronic kidney disease; COPD, chronic obstructive pulmonary disease; CVD, cerebrovascular disease; dias., diastolic; dys., dysfunction, eGFR, estimated glomerular filtration rate; HbA1c, glycated hemoglobin; HF, heart failure; IASD, implanted interatrial shunt device; IQR, interquartile range; LAVI, left atrial volume index; LVEF, left ventricular ejection fraction; MI, myocardial infarction; MRA, mineralocorticoid receptor antagonist; NT-proBNP, N-terminal pro-brain natriuretic peptide; NYHA, New York Heart Association; OACs, oral anticoagulants; PAD, peripheral artery disease; PAH, pulmonary arterial hypertension; PCWP, pulmonary capillary wedge pressure; SGLT-2i, sodium glucose cotransporter-2 inhibitor; sys., systolic; TIA, transient ischemic attack; TR, tricuspid valve regurgitation; vs., versus.

**Table 3:** Sex-dependent characteristics of our HFpEF cohort.

	**Female (N = 109)**	**Male (N = 109)**	***p*-Value**
Demographic data			
Age—years—mean ± SD	75.79 ± 9.50 (N = 109)	74.25 ± 8.78 (N = 108)	0.212
Outpatients—% (a.n.)	62.6 (67) (N = 107)	58.9 (63) (N = 107)	0.578
BMI—kg/m^2^—mean ± SD	28.66 ± 6.86 (N = 108)	28.94 ± 5.06 (N = 108)	0.397
BMI category—median	II (N = 108)	II (N = 108)	0.163
BMI category—% (a.n.)			
I	33.3 (36)	24.1 (26)	
II	28.7 (31)	31.5 (34)	
III	38.0 (41)	44.4 (48)	
BP (sys.)—mmHg—mean ± SD	137.00 ± 28.36 (N = 69)	139.41 ± 23.64 (N = 71)	0.364
BP (dias.)—mmHg—mean ± SD	78.29 ± 14.20 (N = 68)	79.22 ± 11.29 (N = 69)	0.604
Comorbidities			
AF—% (a.n.)	75.2 (82) (N = 109)	73.4 (80) (N = 109)	0.758
AHT—% (a.n.)	94.5 (103) (N = 109)	93.6 (102) (N = 109)	0.776
Diabetes—% (a.n.)	33.0 (36) (N = 109)	38.5 (42) (N = 109)	0.399
(ex-) nicotine abuse—% (a.n.)	22.0 (24) (N = 109)	45.0 (48) (N = 109)	<0.001 *
CVD—% (a.n.)	6.4 (7) (N = 109)	18.3 (20) (N = 109)	0.007 *
PAD—% (a.n.)	12.8 (14) (N = 109)	23.9 (26) (N = 109)	0.036 *
CHD—% (a.n.)	34.9 (38) (N = 109)	52.3 (57) (N = 109)	0.009 *
MI—% (a.n.)	9.2 (10) (N = 109)	16.5 (18) (N = 109)	0.106
Stroke/TIA—% (a.n.)	8.3 (9) (N = 109)	18.3 (20) (N = 109)	0.028 *
COPD—% (a.n.)	19.3 (21) (N = 109)	17.4 (19) (N = 109)	0.728
Sleep apnea—% (a.n.)	9.2 (10) (N = 109)	20.2 (22) (N = 109)	0.022 *
PAH—% (a.n.)	17.4 (19) (N = 109)	25.7 (28) (N = 109)	0.140
CKD—% (a.n.)	56.0 (61) (N = 109)	56.0 (61) (N = 109)	1.000
Comorbidities—No.—mean ± SD	3.67 ± 1.62 (N = 109)	4.38 ± 1.74 (N = 109)	0.002 *
Clinical performance			
NYHA class—median	III (N = 103)	II–III (N = 102)	0.066
NYHA class—% (a.n.)			
I	5.8 (6)	7.8 (8)	
I–II	1.0 (1)	5.9 (6)	
II	25.2 (26)	29.4 (30)	
II–III	16.5 (17)	15.7 (16)	
III	34.0 (35)	30.4 (31)	
III–IV	8.7 (9)	3.9 (4)	
IV	8.7 (9)	6.9 (7)	
Edema—% (a.n.)	32.0 (31) (N = 97)	42.0 (42) (N = 100)	0.146
HFA-PEFF scoring total—median (IQR)	5.00 [2] (N = 105)	5.00 [2] (N = 108)	0.839
HFA-PEFF sc.—% (a.n.)			
1	1.9 (2)	0.9 (1)	
2	5.7 (6)	5.6 (6)	
3	6.7 (7)	13.0 (14)	
4	24.8 (26)	25.0 (27)	
5	29.5 (31)	25.0 (27)	
6	30.5 (32)	30.6 (33)	
H_2_FPEF scoring total -median (IQR)	6.00 [2] (N = 107)	6.00 [2] (N = 107)	0.850
H_2_FPEF scoring—% (a.n.)			
1	0.0 (0)	0.0 (0)	
2	4.7 (5)	1.8 (2)	
3	10.3 (11)	11.9 (13)	
4	8.4 (9)	7.3 (8)	
5	13.1 (14)	21.1 (23)	
6	19.6 (21)	16.5 (18)	
7	23.4 (25)	18.3 (20)	
8	15.9 (17)	13.8 (15)	
9	4.7 (5)	9.2 (10)	
Laboratory findings			
Sodium—mmol/L—mean ± SD	140.19 ± 9.23 (N = 95)	139.39 ± 4.68 (N = 99)	0.447
Potassium—mmol/L—mean ± SD	4.36 ± 0.51 (N = 95)	4.43 ± 0.62 (N = 99)	0.440
Urea—mg/dL—mean ± SD	26.36 ± 17.30 (N = 77)	26.87 ± 12.97 (N = 79)	0.836
Creatinine—mg/dL—mean ± SD	1.28 ± 0.58 (N = 95)	1.60 ± 1.21 (N = 99)	0.022 *
eGFR—mL/min/1.73 m^2^—mean ± SD	48.80 ± 20.73 (N = 92)	51.73 ± 19.94 (N = 94)	0.327
NT-proBNP—pg/mL—median (IQR)	1370.50 (2173) (N = 92)	1095.00 (1706) (N = 94)	0.509
Hemoglobin—g/dL—mean ± SD	12.03 ± 1.78 (N = 95)	12.58 ± 2.07 (N = 99)	0.042 *
Hematocrit—%—mean ± SD	36.72 ± 4.82 (N = 95)	38.63 ± 7.56 (N = 99)	0.036 *
HbA1c—%—mean ± SD	7.60 ± 0.71 (N = 2)	10.60 (N = 1)	0.179
Medical therapy			
ACEi—% (a.n.)	26.6 (29) (N = 109)	33.0 (36) (N = 109)	0.302
ARB—% (a.n.)	43.5 (47) (N = 108)	36.7 (40) (N = 109)	0.308
ARNI—% (a.n.)	3.7 (4) (N = 109)	8.3 (9) (N = 109)	0.154
ARNI/ACEi/ARB—% (a.n.)	73.1 (79) (N = 108)	77.1 (84) (N = 109)	0.507
Beta blockers—% (a.n.)	80.7 (88) (N = 109)	78.9 (86) (N = 109)	0.737
CCB—% (a.n.)	35.8 (39) (N = 109)	36.7 (40) (N = 109)	0.889
Diuretics—% (a.n.)	83.5 (91) (N = 109)	76.1 (83) (N = 109)	0.179
MRA—% (a.n.)	25.7 (28) (N = 109)	21.1 (23) (N = 109)	0.426
SGLT-2i—% (a.n.)	10.1 (11) (N = 109)	14.7 (16) (N = 109)	0.306
ASA—% (a.n.)	17.6 (19) (N = 108)	24.8 (27) (N = 109)	0.198
Statins—% (a.n.)	47.7 (52) (N = 109)	68.8 (75) (N = 109)	0.001 *
OAC—% (a.n.)	71.6 (78) (N = 109)	72.5 (79) (N = 109)	0.881
Sys. HF treatment—% (a.n.)	14.8 (16) (N = 108)	20.2 (22) (N = 109)	0.746
Device therapy			
IASD—% (a.n.)	4.6 (5) (N = 109)	8.3 (9) (N = 109)	0.271
Echocardiographic findings			
LVEF—%—mean ± SD	57.20 ± 5.40 (N = 109)	55.88 ± 5.36 (N = 109)	0.072
LAVI—mL/m^2^—mean ± SD	47.85 ± 17.93 (N = 91)	49.66 ± 19.88 (N = 94)	0.516
E/e’—mean ± SD	15.34 ± 5.87 (N = 78)	12.91 ± 4.56 (N = 86)	0.006 *
E/A—mean ± SD	1.76 ± 0.95 (N = 63)	1.8 ± 1.02 (N = 72)	0.792
e’ septal—cm/s—mean ± SD	7.05 ± 3.40 (N = 36)	7.15 ± 4.10 (N = 44)	0.906
e’ lateral—cm/s—mean ± SD	9.24 ± 3.20 (N = 40)	9.65 ± 3.43 (N = 46)	0.565
TR velocity—m/s—mean ± SD	3.02 ± 0.53 (N = 26)	3.22 ± 0.49 (N = 25)	0.174
Degree dias. dys.—total—median	II (N = 63)	II (N = 69)	0.704
Degree dias. dys.—% (a.n.)			
I	20.6 (13)	23.2 (16)	
I–II	0.0 (0)	1.4 (1)	
II	42.9 (27)	39.1 (27)	
II–III	1.6 (1)	4.3 (3)	
III	34.9 (22)	31.9 (22)	
Invasive hemodynamics			
CI—L/min/m^2^—mean ± SD	2.37 ± 0.54 (N = 32)	2.33 ± 0.86 (N = 33)	0.305
PCWP—mmHg—mean ± SD	19.36 ± 5.77 (N = 28)	20.94 ± 8.37 (N = 33)	0.925

Average data shown as mean ± SD. Numbers followed by square brackets are median with interquartile range. Categorical variables are described by percentage and absolute number (a.n.). Colored rows indicate subcategories. Significant differences are indicated by an asterisk. ACEi, angiotensin-converting enzyme inhibitor; AHT, arterial hypertension; AF, atrial fibrillation; ARB, angiotensin receptor blocker; ARNI, angiotensin receptor–neprilysin inhibitor; ASA, acetylsalicylic acid; BMI, body mass index; BP, blood pressure; CCB, calcium channel blocker; CHD, coronary heart disease; CI cardiac index; COPD, chronic obstructive pulmonary disease; CVD, cerebrovascular disease; dias., diastolic; dys., dysfunction, eGFR, estimated glomerular filtration rate; HbA1c, glycated hemoglobin; HF, heart failure; IASD, implanted interatrial shunt device; IQR, interquartile range; LAVI, left atrial volume index; LVEF, left ventricular ejection fraction; MI, myocardial infarction; MRA, mineralocorticoid receptor antagonist; NT-proBNP, N-terminal pro-brain natriuretic peptide; NYHA, New York Heart Association; OACs, oral anticoagulants; PAD, peripheral artery disease; PAH, pulmonary arterial hypertension; PCWP, pulmonary capillary wedge pressure; SGLT-2i, sodium glucose cotransporter-2 inhibitor; sys., systolic; TIA, transient ischemic attack; TR, tricuspid valve regurgitation.

## Data Availability

As our data include sensitive information (patient data), they are unavailable to access.

## Supplementary Materials

The following supporting information can be downloaded at: https://www.mdpi.com/article/10.3390/jcm14103348/s1, Table S1: Correlation of clinical variables with sex and BMI.

## Abbreviations

ACEi	Angiotensin-Converting Enzyme Inhibitor
AF	Atrial Fibrillation
AHT	Arterial Hypertension
ANOVA	Analysis of Variance
ARB	Angiotensin Receptor Blocker
ARNI	Angiotensin Receptor–Neprilysin Inhibitor
ASA	Acetylsalicylic Acid
BMI	Body Mass Index
CCB	Calcium Channel Blocker
CHD	Coronary Heart Disease
CI	Cardiac Index
CKD	Chronic Kidney Disease
COPD	Chronic Obstructive Pulmonary Disease
CVD	Cerebrovascular Disease
EF	Ejection Fraction
eGFR	estimated Glomerular Filtration Rate
ESC	European Society of Cardiology
HbA1c	Glycated Hemoglobin
HF	Heart Failure
HFA-PEFF	Heart Failure Association Pre-test Assessment Echo, Function, and Final Etiology
HFpEF	Heart Failure with preserved Ejection Fraction
IASD	Interatrial Shunt Device
LAVI	Left Atrial Volume Index
LV	Left Ventricular
LVEF	Left Ventricular Ejection Fraction
NT-proBNP	N-terminal pro-brain natriuretic peptide
MI	Myocardial Infarction
MRA	Mineralocorticoid Receptor Antagonist
NYHA	New York Heart Association
OACs	Oral Anticoagulants
PAD	Peripheral Artery Disease
PAH	Pulmonary Arterial Hypertension
PCWP	Pulmonary Capillary Wedge Pressure
TIA	Transient Ischemic Attack
SGLT-2i	Sodium Glucose Cotransporter-2 Inhibitor
SPSS	Statistical Package for the Social Sciences
SQL	Structured Query Language
