# Supplementary material for: Obesity and Comorbidities in HFpEF: A Retrospective Cohort Analysis in a University Hospital Setting
Source: J Clin Med. 2025 May 12;14(10):3348. doi: 10.3390/jcm14103348 (PMC12112532; doi:10.3390/jcm14103348)
Supplement: Supplementary file 1 [file jcm-14-03348-s001.zip › jcm-3580424-supplementary.pdf]

Supplementary Materials

Table S1: Correlation of clinical variables with sex and BMI.

	**Sex**<br>r [0.95% confidence interval]	**BMI**<br>r [0.95% confidence interval]
Age		-0.201 [-0.33; -0.065]
Edema		0.233 [0.097; 0.365]
eGFR		-0.155 [-0.297; -0.006]
NYHA		0.153 [0.012; 0.288]
Hemoglobin	0.146 [0.001; 0.285]	
Creatinine	0.164 [0.024; 0.298]	
Haematocrit	0.149 [0.009; 0.284]	
(ex-) nicotine abuse	0.241 [0.108; 0.366]	
AHT		0.136 [-0.002; 0.268]
Diabetes		0.326 [0.198; 0.444]
CVD	0.181 [0.045; 0.310]	
PAD	0.142 [0.006; 0.274]	
CHD	0.176 [0.040; 0.305]	
Stroke/TIA	0.149 [0.012; 0.280]	
Sleep apnea	0.156 [0.019; 0.286]	0.281 [0.149; 0.403]
Comorbidities	0.192 [0.057; 0.321]	
ARNI		0.137 [-0.001; 0.269]
CCB		0.173 [0.037; 0.303]
Diuretics		0.189 [0.053; 0.318]
MRA		0.173 [0.037; 0.304]
SGLT-2i		0.237 [0.103; 0.362]
Statins	0.214 [0.080; 0.341]	
E/e’	-0.228 [-0.368; -0.077]	

Correlations are described by correlation coefficient, r; the 95% confidence interval is given in the brackets. AHT, arterial hypertension; ARNI, angiotensin receptor–neprilysin inhibitor; CCB, calcium channel blocker; CHD, coronary heart disease; CVD, cerebrovascular disease; eGFR, estimated glomerular filtration rate; MRA, mineralocorticoid receptor antagonist; NYHA, New York Heart Association; PAD, peripheral artery disease; r, correlation coefficient; SGLT-2i, sodium glucose cotransporter-2 inhibitor; TIA, transient ischemic attack.
